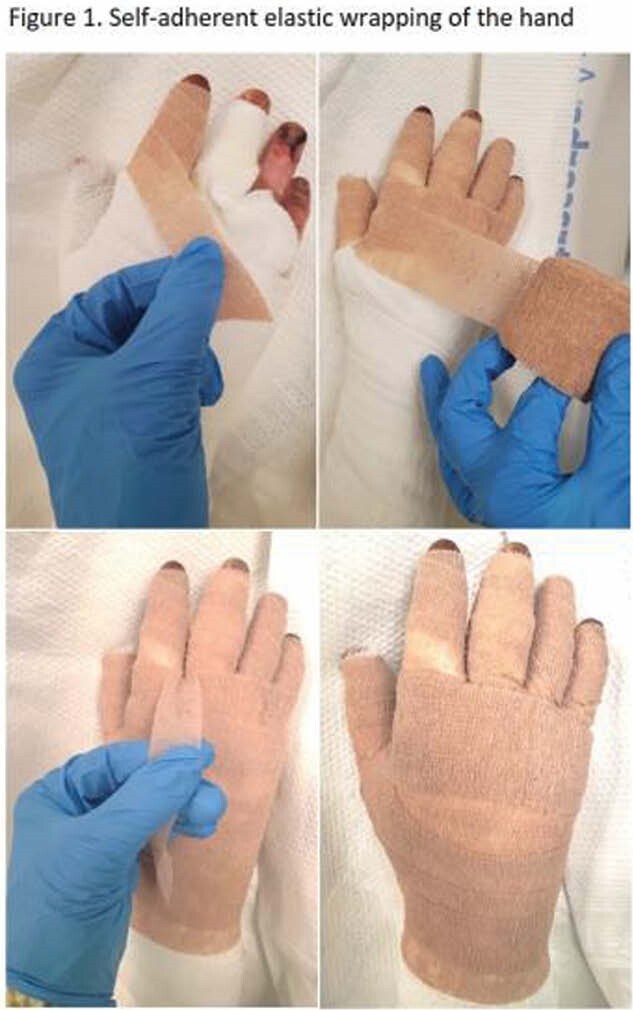# 81 Post-operative Self-adherent Compression Wrapping of the Hand and Its Impact on Skin-graft Viability

**DOI:** 10.1093/jbcr/irac012.084

**Published:** 2022-03-23

**Authors:** Scott F Vocke, Joseph S Puthumana, Brooke Dean, Gregory Andre, Joshua Rodriguez, Misao Mercadante, Charles S Hultman, Scott Lifchez, Qingwen Kawaji, Sohayla Rostami, Stephanie L Martinez, Julie Caffrey

**Affiliations:** Johns Hopkins, Baltimore, Maryland; Johns Hopkins University Department of Plastic and Reconstructive Surgery, Baltimore, Maryland; Johns Hopkins Bayview Medical Center, Baltimore, Maryland; Johns Hopkins Bayview Medical Center, Lutherville Timonium, Maryland; Johns Hopkins Bayview, Timonium, Maryland; Johns Hopkins Bayview, Baltimore, Maryland; Johns Hopkins University School of Medicine, Baltimore, Maryland; Johns Hopkins, Baltimore, Maryland; Johns Hopkins, Baltimore, Maryland; Johns Hopkins University, Baltimore, Maryland; Johns Hopkins, Towson, Maryland; Johns Hopkins, Baltimore, Maryland

## Abstract

**Introduction:**

Potential complications of autografting for burn wound coverage of the hand include edema, hematoma formation, and bleeding; all of which can lead to graft failure. Self-adherent elastic wraps are commonly used by burn rehabilitation clinicians to minimize complications by providing graft protection and decreasing edema post-operatively; however, there is a lack of evidence on its impact on graft healing. The purpose of this study was to determine if the application of self-adherent elastic wraps to the hand in the operating room after autografting improves the percentage of graft viability.

**Methods:**

A retrospective chart review was performed for 37 patients with burned hands who underwent autografting from January 2017 to July 2021. Grafted hands were categorized into 2 groups: post-operative dressings with and without self-adherent elastic wraps. Post-operative day 4 pictures for both groups were collected from the medical record and a blinded digital photograph analysis of graft viability was performed by 5 expert raters including 3 Burn Surgery Fellows,1 Burn Attending Surgeon and 1 Hand Attending Surgeon. A rating system was developed based on percentage of graft take as seen in Table 1 and presence of hematomas were assessed.

**Results:**

Patients who received self-adherent elastic wraps suffered burns with significantly larger TBSA (p=0.007) and were admitted for a longer duration (p=0.009) than patients who did not. Patients with elastic wrap had a higher percentage of grafts with >95% take (64.0% vs 41.7%, p=0.227) and a lower rate of hematoma formation (24.0% vs. 41.7%, p=0.443). Intra-class correlation coefficient across raters was 0.90 for graft take and 0.87 for determining presence of hematomas, indicating excellent interrater reliability.

**Conclusions:**

Despite suffering larger burns requiring longer hospitalizations, patients who received elastic wrap had a higher rate of >95% graft take than those without. This study is limited by a relatively small sample size, however these findings warrant continued research in the use of self-adherent elastic wrap to maximize graft take in hand burns.